# Quantum Spin‐1/2 Dimers in a Low‐Dimensional Tetrabromocuprate Magnet

**DOI:** 10.1002/chem.202200855

**Published:** 2022-04-27

**Authors:** Gavin Sampson, Nicholas C. Bristowe, Sam T. Carr, Asad Saib, Gavin B. G. Stenning, Ewan R. Clark, Paul J. Saines

**Affiliations:** ^1^ School of Physical Sciences University of Kent Canterbury Kent CT2 7NH UK; ^2^ Centre for Materials Physics Durham University South Road Durham DH1 3LE UK; ^3^ ISIS Neutron and Muon Source Rutherford Appleton Laboratory Harwell Science and Innovation Campus Didcot OX11 0QX UK

**Keywords:** cuprates, density functional calculations, halides, magnetic properties, X-ray diffraction

## Abstract

This work describes a homometallic spin‐1/2 tetrabromocuprate adopting a bilayer structure. Magnetic‐susceptibility measurements show a broad maximum centred near 70 K, with fits to this data using a Heisenberg model consistent with strong antiferromagnetic coupling between neighbouring copper atoms in different layers of the bilayer. There are further weak intralayer ferromagnetic interactions between copper cations in neighbouring dimers. First‐principles calculations are consistent with this, but suggest there is only significant magnetic coupling within one direction of a layer; this would suggest the presence of a spin ladder within the bilayer with antiferromagnetic rung and weaker ferromagnetic rail couplings.

## Graphical abstract

**Only one way**: (3,4‐lutH)_2_CuBr_4_ (3,4‐lut=3,4‐lutidine) is a homometallic spin‐1/2 tetrabromocuprate that adopts a bilayer structure with strong antiferromagnetic coupling between dimers across the bilayer and weaker intralayer ferromagnetic interactions. First‐principles calculations suggest there is only significant magnetic coupling within one direction of a layer; this is consistent with a spin‐ladder motif with antiferromagnetic rung and weaker ferromagnetic rail couplings.

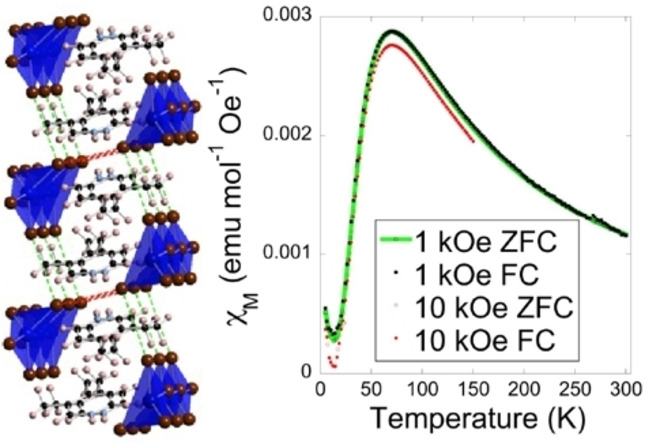

## Introduction

Low‐dimensional magnets have long been a playground for discovering unconventional physics.[^1^, ^2^, ^3^] From an experimental point of view, ideal systems have strong magnetic coupling in one‐ or two‐dimensional units, which are well isolated from each other. This includes magnetic chains, which are hosts to spinon quasi‐particles that fractionalise electrons by carrying their spin but not charge,[^4^, ^5^] and magnetic sheets, which when doped provide models for electronic behaviour in high temperature superconductors.^[6]^ Spin ladders, which are formed of two or more interconnected chains and often have spin liquid ground states, offer insight into phenomena at the borderline of one‐ and two‐dimensional magnetic systems.[^7^, ^8^] Magnetic bilayers are geometrically analogous to spin ladder but are at the interface of two and three dimensions; intrinsic magnetic bilayers are rare with only a handful known.[^9^, ^10^, ^11^, ^12^, ^13^, ^14^, ^15^] In contrast intrinsic spin ladders with antiferromagnetic coupling both within their chains, commonly referred to as legs or rails, and between them, the rungs, have been relatively well studied,^[16]^ but those with ferromagnetic coupling within these units are far less so.[^17^, ^18^] There are a handful of theoretical studies predicting a variety of phases,[^19^, ^20^, ^21^, ^22^] including rung singlet, spin Luttinger liquid, and stripe‐ferromagnetic phases depending on model parameters.

Amongst low‐dimensional magnets, spin‐1/2 compounds are particularly of interest due to the highly quantum nature associated with low spins. When developing novel low‐dimensional magnets the use of spin‐1/2 inorganic centres provides stronger magnetic coupling and greater chemical stability. The well explored metal oxides, however, typically adopt close‐packed structures that do not have sufficient spacing between chains and sheets to eliminate residual coupling between these units.[^23^, ^24^] Searching for new magnetic materials containing both organic and inorganic building blocks provides an alternative route to realising well isolated, low‐dimensional units because of the unique architectures they adopt to accommodate their nonspherical molecular components.[^25^, ^26^, ^27^] With respect to magnetic bilayers, the only previous examples of spin‐1/2 magnetic bilayer materials built from combining inorganic and organic building blocks are the (tetrenH_5_)_0.8_Cu_4_[B(CN)_8_]_4_ and (dienH_3_)Cu_4_[B(CN)_8_]_4_ compounds (where tetren is tetraethylenepentamine, dien is diethylenetriamine and B is Mo or W),[^10^, ^11^] which are complicated by having two distinct magnetic ions in their bilayers and undergoing transitions from antiferromagnetic to ferromagnetic behaviour under the application of very modest applied fields.^[28]^ The closest approximation to a spin ladder containing only ferromagnetic rail coupling is a [{CuCl(*O*‐2‐methylisothiazole‐3‐one}_2_(μ‐Cl)_2_] complex but this has competing antiferromagnetic interactions diagonally across the ladder comparable in strength to the rung and rail interactions, a significant departure from an ideal spin ladder.^[29]^ This leaves spin ladders with purely ferromagnetic rail coupling almost entirely restricted to one family of organic magnets based on verdazyl radicals, with no known spin‐ladder compounds known based on inorganic magnetic centres.[^30^, ^31^, ^32^, ^33^, ^34^, ^35^]

While much of the interest in inorganic–organic magnets focuses on coordination polymers and metal–organic frameworks, in which the organic building blocks link neighbouring magnetic centres,[^36^, ^37^] alternative materials for such studies include those where the organic components act as scaffolding around which inorganic frameworks are structured. This offers the potential for shorter magnetic coupling pathways that strengthen magnetic coupling. Amongst such materials, the versatile A_2_MX_4_ (where A is a monovalent organic cation, M a divalent transition metal and X a halide) tetrahalometallates have already attracted significant attention for their ability as low‐dimensional hosts, including for magnetic chains,[^38^, ^39^] sheets[^40^, ^41^] and ladders.[^42^, ^43^, ^44^, ^45^] These materials are typically built from molecular MX_4_ units with magnetic coupling occurring through halide–halide contacts. While the separation of their low‐dimensional magnetic units by bulky organic cations insulate these from each other, short magnetic coupling pathways between these units are commonly found in these materials. This enables them to exhibit much stronger low‐dimensional magnetic interactions than found in purely organic magnets or other materials built from combining inorganic and organic building blocks, such as metal–organic frameworks.

In this paper, we report a tetrabromocuprate incorporating protonated 3,4‐lutidine (3,4‐lutH) cations that adopts a bilayer structure. (3,4‐lutH)_2_CuBr_4_ contains two layers of Cu centres in close proximity to each other, allowing magnetic communication between them, with neighbouring bilayers well separated by 3,4‐lutH cations. Magnetic property measurements indicate a transition temperature above 70 K arising from dominant antiferromagnetic coupling between nearest neighbours in these different layers with intralayer ferromagnetic coupling. Intriguingly, however, first principles calculations indicate that significant coupling only occurs along one axis of the layers, potentially leading to hidden spin‐1/2 ladders in this compound with rare ferromagnetic rail coupling. Although it is not possible to clearly determine experimentally whether (3,4‐lutH)_2_CuBr_4_ is a spin ladder or bilayer through analysing its bulk properties either result would be a unique spin‐1/2 homometallic magnetic material.

## Results and Discussion

### Crystal structure

**Figure 1:**
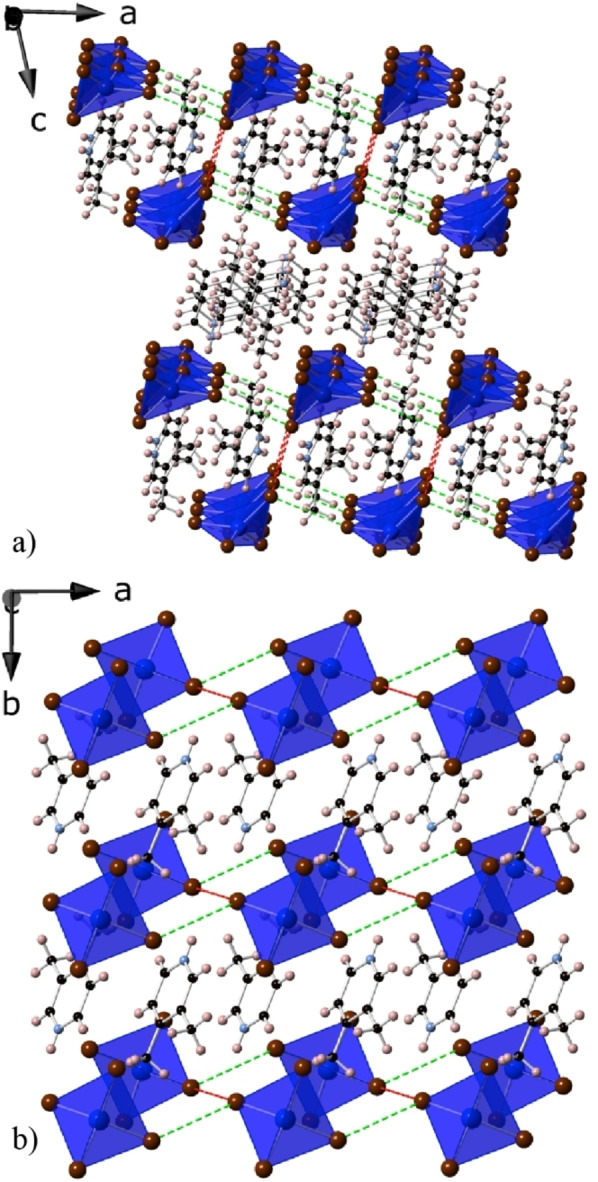
Crystal structure of (3,4‐lutH)_2_CuBr_4_ showing a) a view of the stacking of the bilayers along the *b*‐axis and b) the two layers in a single bilayer. The Cu tetrahedra are dark blue; bromide, carbon, nitrogen and hydrogen atoms are shown as maroon, black, light blue and pink spheres, respectively. The dominant magnetic coupling through Br⋅⋅⋅Br contacts, *J* and *J*_N1_, indicated by DFT are shown as red and light green dotted lines.

Crystals of (3,4‐lutH)_2_CuBr_4_ suitable for X‐ray diffraction structure determination were readily prepared by vapour diffusion of diethyl ether into an ethanolic solution of 3,4‐lut and CuBr_2_ ⋅ 2 H_2_O acidified with HBr. The asymmetric unit of the triclinic structure of (3,4‐lutH)_2_CuBr_4_ contains one CuBr_4_ complex, with four distinct Br atoms, and two complete 3,4‐lutH cations (see Figure S1 in the Supporting Information for asymmetric unit and Table S1 for crystallographic details). The Cu centres are packed into a square lattice with neighbouring Cu ions in the two layers in a bilayer slightly offset from each other (Figure 1). Neighbouring bilayers are directly stacked on top of each other. One of the distinct 3,4‐lutH cations sits in space within the bilayer while the other sits between them. The Cu adopts a tetrahedral environment with very similar Cu−Br bond distances but bond angles heavily distorted from an ideal tetrahedra (see Table S2 for selected bond distances and angles). The Cu centre was found to have a bond valence sum of 2.10, consistent with Cu being divalent.^[46]^

As in other tetrahallometallates, magnetic exchange is expected to occur through the short Cu−Br⋅⋅⋅Br−Cu pathways with Br⋅⋅⋅Br distances playing a key role in determining magnetic coupling strength as these are more variable than the Cu−Br distances.[^42^, ^43^] The shortest Cu−Br⋅⋅⋅Br−Cu pathways along both axes of a layer have very similar distances, 9.195(10) and 9.187(8) Å, for the *a*‐axis and *b*‐axis, respectively, with a shortest Br⋅⋅⋅Br distance of 4.408(7) and 4.429(5) Å along these directions. In contrast, the Cu−Br⋅⋅⋅Br−Cu distance between layers in a bilayer is much shorter at 8.508(13) Å, with a Br⋅⋅⋅Br distance of 3.739(8) Å. The Cu−Br⋅⋅⋅Br bond angles in these pathways are 106.42(5)° and 136.346(15)° along the *a*‐axis, 122.035(18)° and 141.70(5)° along the *b*‐axis and 149.74(5)° along the *c*‐axis. There is a greater difference, however, between the Cu−Br⋅⋅⋅Br−Cu torsion angles within and between the layers with angles of 134.64(2)° along the *a*‐axis, 122.13(2)° along the *b*‐axis and 180° along the *c*‐axis. The closest Cu−Br⋅⋅⋅Br−Cu distance between neighbouring bilayers is much longer at 11.254(17) Å; this involves a Br⋅⋅⋅Br contact more than 2.5 times the intra‐bilayer separations and it is therefore expected that the magnetic coupling between adjacent layers should be negligible. As discussed below, this is confirmed by DFT calculations.

The formation of a sample of (3,4‐lutH)_2_CuBr_4_ suitable for bulk analysis was achieved by recrystallisation of the crude product using an ethanolic solution acidified by HBr with precipitation initiated by vapour diffusion using diethyl ether. This was confirmed by a Le Bail fit, carried out using the programme Rietica,^[47]^ using the unit cell obtained from single‐crystal studies with only trace quantities of an unidentified impurity observed (Figure S2). The purity of this sample was further confirmed by elemental analysis results (experimental values C 27.88 %, H 3.50 % and 4.55 % N *c.f*. to calculated values of 28.01, 3.36 and 4.67 %, respectively). Although the structure and magnetic properties of this material have not been previously reported, there is a previous report of a (3,4‐lutH)_2_CuBr_4_ phase reported to have a similar dark purple colour and an ambient temperature effective magnetic moment of 1.79 μ_B_, similar to the value reported herein, which may suggest this is not the first time this material has been made.^[48]^

### Physical property measurements

Thermogravimetric analysis showed that the compound was stable until 135 °C in both air and nitrogen, thus suggesting decomposition is a result of thermal instability and that the materials are relatively chemical stable in air (Figure S3). Above this temperature the material decomposes in a two‐stage process firstly between 135 and 330 °C in an endothermic process followed by further weight loss above 400 °C in an exothermic process.

**Figure 2:**
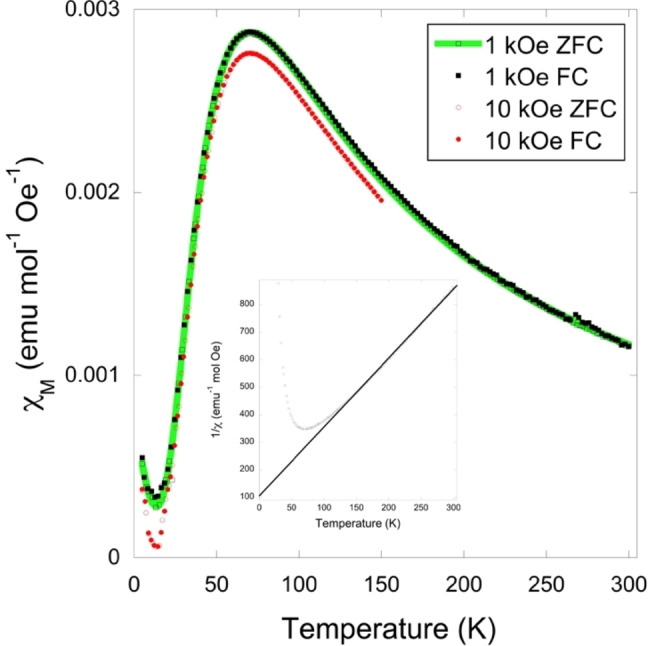
Plot of the magnetic susceptibility of (3,4‐lutH)_2_CuBr_4_ vs. temperature at 1 and 10 kOe with ZFC and FC measurements shown by empty and filled symbols, respectively: The 1 and 10 kOe data are both shown across the full range measured. The fit of the magnetic model described in the text is shown in green. The insert shows a Curie–Weiss fit to inverse susceptibility vs. temperature above 120 K.

**Figure 3:**
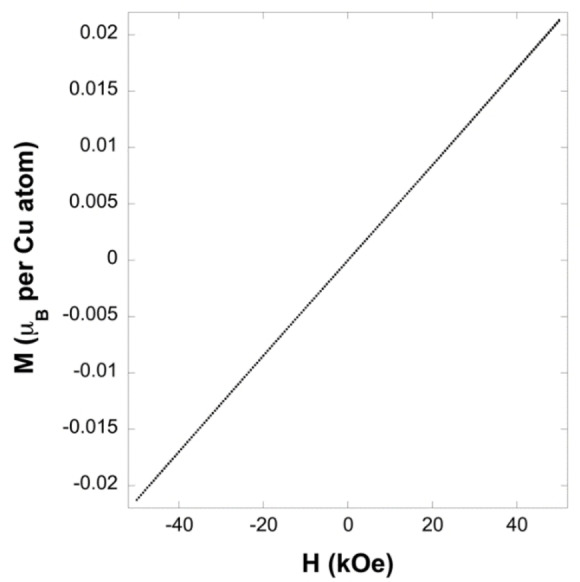
Isothermal magnetisation of (3,4‐lutH)_2_CuBr_4_ at 45 K.

DC magnetic susceptibility measurements between 2 and 300 K in 1 and 10 kOe applied fields both revealed broad maxima centred at 71 K, consistent with short range magnetic order (Figure 2). Zero‐field‐cooled (ZFC) and field‐cooled (FC) susceptibility measurements do not diverge, consistent with strong, low‐dimensional antiferromagnetic coupling. Below 14 K, susceptibility increases again, likely due to the presence of a small quantity of an unknown paramagnetic impurity, which might also be responsible for two very weak peaks in the powder X‐ray diffraction pattern. Above 120 K, the sample behaves as a Curie–Weiss paramagnet with fits to a 1 kOe ZFC measurement above this temperature indicating a Weiss temperature of −42.2 K; this is consistent with dominant antiferromagnetic coupling (Figure 2, insert). This also yields an effective magnetic moment of 1.79 μ_B_, similar to the expected spin‐only magnetic moment of spin‐1/2 Cu^2+^, 1.73 μ_B_. *χT* is 0.347 emu K mol^−1^ Oe^−1^ at 300 K in a 1 kOe field, below the value of 0.375 emu K mol^−1^ Oe^−1^ expected for an isolated paramagnet, thus suggesting the existence of weak antiferromagnetic coupling at ambient temperature. *χT* decreases rapidly below 150 K and becomes vanishingly small below 10 K consistent with the presence of strong antiferromagnetic coupling (Figure S4). An isothermal magnetisation measurement at 45 K indicates that the sample magnetisation increases in a linear fashion with applied field reaching 0.022 μ_B_ per Cu atom at an applied field of 50 kOe, far from the saturation value expected for Cu^2+^, consistent with strong antiferromagnetic coupling (Figure 3). Isothermal magnetisation measurements obtained at lower temperatures are broadly consistent with those shown in Figure 3, although the more gradual slope as antiferromagnetic coupling becomes stronger relative to thermal motion leads to these measurements becoming quite noisy as temperature decreases.

AC susceptibility measurements with an AC drive field of 3 Oe, performed in both the absence of a DC field and a 50 Oe DC field, lack any frequency dependence in *χ′* and any significant signal in *χ′′* (Figures S5 and S6). This suggests a lack of magnetic dynamics consistent with strongly coupled antiferromagnetic dimers forming near 70 K.

Allowing for the observed paramagnetic impurity, if the magnetic properties of (3,4‐lutH)_2_CuBr_4_ are dominated by dimer coupling along the *c*‐axis and its interactions are Heisenberg‐like, as expected for Cu^2+^, *χ* is expected to be of the form:

$$\chi =\left[4C/T\right]/\left[3+exp\left(-J/T\right)-J{}^{'}/T\right]+C_{imp}/\left[T-\theta_{imp}\right]$$

where *C* and *C*_imp_ are the Curie constants of the (3,4‐lutH)_2_CuBr_4_ and the paramagnetic impurity, *θ*_imp_ is the Weiss temperature of the impurity, *J* is the intradimer coupling strength, *J’* is a parameterisation of the interdimer coupling strength, and *T* is temperature (see the Supporting Information for derivation). An excellent fit was obtained to the 1 kOe ZFC data with this model with values of *C*=0.3755(15) emu K mol^−1^ Oe^−1^, *C*_imp_=0.0044(3) emu K mol^−1^ Oe^−1^, *J*=−114.4(2) K, *J’*=35(3) K and *θ*_imp_ is −3.5(8) K (Figure 2). While this involves a significant number of parameters, the inclusion of the impurity is essential to capture the increase in susceptibility below 15 K, although we do not ascribe significant physical meaning to the value of *θ*_imp_ obtained. Fits without the interdimer coupling *J’* replicate the general shape of the peak but the fit is significantly poorer with a significant underestimation of *χ* between the transition temperature and 130 K, leading to a doubling of the *χ* ^2^ measure of fit. This result indicates that the antiferromagnetic coupling along the *c*‐axis dominates the magnetic properties. Given the geometry of the system *J’* can then be interpreted as indicating weaker ferromagnetic intralayer coupling, we note the magnitude obtained is an underestimation of the actual interaction strength and that it is not possible to distinguish whether the interactions within the layer are one or two dimensional from this fit. The dominant coupling between dimers is not surprising given the shorter Cu−Br⋅⋅⋅Br−Cu pathways between these. Somewhat more surprising is the ferromagnetic coupling within the individual layers, as this is only rarely seen in A_2_CuX_4_ compounds.[^39^, ^49^, ^50^] Indeed the strength of the ferromagnetic coupling found here is, to the best of our knowledge, unprecedented amongst A_2_CuX_4_ compounds where magnetic coupling occurs via two halide bridges. We would, however, add a note of caution that, as the Weiss temperature is the only measure of the magnetic coupling in many of these materials such ferromagnetic coupling would be overlooked in cases where stronger antiferromagnetic coupling is present, since that would lead to a negative Weiss temperature, as is indeed the case in (3,4‐lutH)_2_CuBr_4_. There is no clear trend amongst those compounds exhibiting ferromagnetic coupling and their Cu−Br⋅⋅⋅Br or Cu−Br⋅⋅⋅Br−Cu angles. The large difference in the torsion angles within and between the layers in a structural bilayer is not likely to cause this as the well‐studied ((CH_3_)_2_NH_2_)(3,5‐lutH)CuX_4_ (X=Cl or Br) spin ladders have antiferromagnetic rail and rung coupling despite torsion angles close to 90° and 180°, respectively.[^42^, ^51^]

### Density functional theory (DFT) calculations

**Figure 4:**
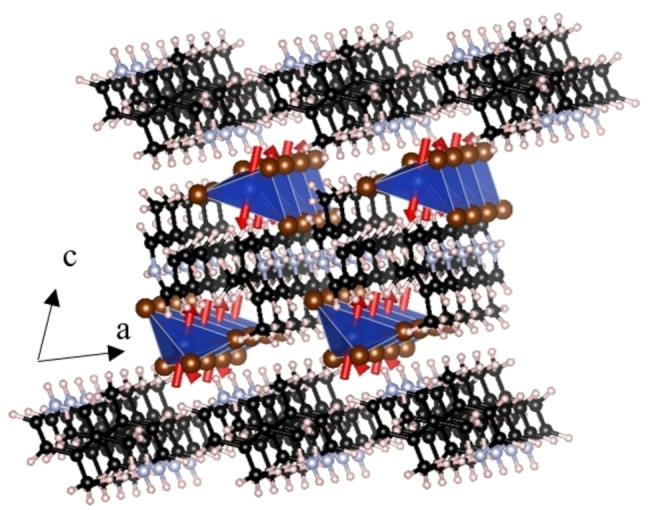
Depiction of AFM3, the lowest‐energy collinear magnetic structure calculated by DFT. It features antiferromagnetic *J* and *J*_N2_ and ferromagnetic *J*_N1_ magnetic coupling. The spins are arbitrarily depicted along the *c*‐axis, as collinear DFT calculations do not consider spin orientation.

During first principles calculations, the structure was initially relaxed with antiferromagnetic Cu intra‐dimer spins with ferromagnetic coupling between dimers (labelled AFM1); this gave good structural agreement with X‐ray diffraction (Tables S1 and S2). Our optimised, rather than experimental, structure is used to determine exchange couplings to avoid any artefacts arising from uncertainties in the experimental H positions. The energies of six possible magnetic ordering patterns were then considered, labelled FM and AFM1‐5 (see Table S3 for energies and Figure S7 for configurations). FM corresponds to ferromagnetic ordering of all Cu moments. AFM1 corresponds to antiferromagnetic interactions between the two Cu ions in the unit cell (and ferromagnetic orderings between these and all neighbouring cells). AFM2 also corresponds to antiferromagnetic interactions between the two Cu ions in the unit cell, but these are anti‐aligned between neighbouring cells along the *a*‐axis (and ferromagnetic orderings between these and neighbouring cells along the *b*‐ and *c*‐axes). Similarly, AFM3 has antiferromagnetic interactions within the unit cell and also between neighbouring cells along the *b*‐axis (but ferromagnetic coupling between neighbouring cells along *a* and *c*); this was found to be the lowest energy magnetic state amongst those calculated (Figure 4). AFM4 has antiferromagnetic interactions within the unit cell and between neighbouring cells along both the *a*‐ and *b*‐axes (but ferromagnetic coupling along *c*). AFM5 has antiferromagnetic interactions within the unit cell and also between neighbouring cells along the *c*‐axis (but ferromagnetic coupling between neighbouring cells along *a* and *b*). The resulting energies were then used to determine both the intra‐dimer *J* and four distinct inter‐dimer *J*_N_ as a function of U between 3 and 11 eV (see Table 1 for values and Figures 1 and S8 for diagrams indicating contacts associated with this).

**Table 1:** The change in DFT intradimer *J* and four interdimer *J*_N1–N4_ values with varying U.^[a]^

Magnetic	Energy [K]				
exchange coupling	U=3 eV	U=5 eV	U=7 eV	U=9 eV	U=11 eV
J	−638	−518	−386	−247	−156
J_N1_	319	254	184	112	65
J_N2_	−16	−17	−17	−17	−16
J_N3_	−10	−5	−0.5	4	7
J_N4_	30	34	36	37	37

[a] *J* represents the intradimer interactions, *J*_N1_ the interactions within a layer along the *a*‐axis, *J*_N2_ the interactions within a layer along the *b*‐axis, *J*_N3_ interactions between neighbouring bilayers along the *c*‐axis, and *J*_N4_ an alternative longer diagonal ladder rung interaction.

In agreement with our modelling of the susceptibility data, we find predominant antiferromagnetic intra‐dimer coupling, which are twice as strong as any other interactions essentially regardless of the value of U chosen. Of the four interdimer couplings considered the shortest exchange pathway along the *a*‐axis, *J*_N1_, was consistently found to be strongest. In contrast *J*_N2_, the coupling with a single layer along the *b*‐axis, is determined to be much lower in strength, typically an order of magnitude for most values of U modelled. This indicates highly anisotropic ferromagnetic coupling between dimers. As anticipated from the crystal structure coupling between the bilayers, *J*_N3_, is negligible, approximately two orders of magnitude lower than *J* and *J*_N1_. Thus DFT suggests the magnetic interactions within this material resemble a spin ladder with strong antiferromagnetic rungs, weaker ferromagnetic rails, and inter‐ladder interactions that are at least one order of magnitude weaker. We note that the overestimated magnetic coupling strength determined by our calculations compared to the experimental results is consistent with previous DFT studies,[^53^, ^54^, ^55^] but also that the computed values strongly depend on the value of U utilised (Table S4). Larger values of U produce closer agreement with experiment, however perhaps more importantly, the difference in *J’*/*J* compared to *J_N_/J* is consistent with the deviations expected from mean field theory for a low‐dimensional system. Crucially the qualitative picture of a spin‐ladder is unchanged for all reasonable values of on‐site Coulomb repulsion (Table 1).

The magnetic property measurements in this study indicate (3,4‐lutH)_2_CuBr_4_ is a low‐dimensional magnetic spin‐1/2 system with strong antiferromagnetic dimer coupling and ferromagnetic coupling between dimers. Whereas it would be anticipated from the crystal structure this would be a bilayer the DFT, calculations indicate negligible magnetic coupling along the *b*‐axis, reducing this to a spin ladder with unusual ferromagnetic rails. The formation of dimers between the two layers means that the bulk thermodynamic properties of the system are very weakly dependent on details of the interdimer coupling. Distinguishing between the spin ladder or spin bilayer cases using bulk property measurements would require a magnetic field strong enough to inhibit the dimer formation, whereby details of the other interactions would play a far greater role. Given that the dimer energy is −114.4(2) K, this would require a magnetic field of about 85 T, which is impractical. Alternatively, microscopic studies of the material, principally using inelastic neutron scattering (INS), could be used to confirm the relative strength of magnetic coupling suggested by DFT and more accurately estimate their strength. The absence of a clear route to making large single crystals of this phase and the high hydrogen content in the material combined with extreme difficulty in deuteration, however, make such INS studies extremely challenging and beyond the scope of this initial study.

Although we cannot distinguish experimentally whether (3,4‐lutH)_2_CuBr_4_ is a 1/2 spin ladder or a spin bilayer with ferromagnetic coupling between the dimers, either would be the first inorganic–organic material containing homometallic magnetic centres. On the basis of the DFT results it appears more likely that (3,4‐lutH)_2_CuBr_4_ is an unusual spin‐1/2 ladder with *J’*/*J* of −0.5 and ferromagnetic rail couplings. The only other good spin ladder model compounds with such ferromagnetic coupling are based on 1,3,5‐triphenylverdazyl radicals with the one of the *met*a‐phenyl rings substituted with different halogens at the *ortho*‐ and *para*‐positions, in which *J’*/*J* ranges from 0.6–1.8.[^30^, ^32^] Low‐dimensional order of these radical‐based ladders occurs only below 10 K, with three dimensionally ordered states evolving below 1.5 K in two of these.^[32]^ In contrast, low‐dimensional short range order of (3,4‐lutH)_2_CuBr_4_ is indicated to occur at much higher temperatures, as indicated by a feature in the susceptibility near 70 K, while there is no indication of the emergence of a long range ordered state down to 2 K, the lowest temperature examined in this study. This suggests that (3,4‐lutH)_2_CuBr_4_ likely contains more isolated spin ladders than the known cases with ferromagnetic rail coupling in addition to this state persisting to higher temperatures, which may facilitate its study by a broader range of techniques. Despite the poorer isolation of their spin ladders and some weak diagonal coupling between the ladder rails,^[33]^ the verdazyl radicals have attracted attention as candidates for Tomonga–Luttinger liquids and quasi‐1D Bose–Einstein Condensates, examples of quantum phase transitions.[^34^, ^35^] If (3,4‐lutH)_2_CuBr_4_ can be confirmed experimentally to be a spin ladder increasing *J’*/*J*, through selective chemical replacement of the Br with other halides or application of high pressure or magnetic fields would increase competition between the rail and rung interactions. Understanding how the states in (3,4‐lutH)_2_CuBr_4_ change with the relative strength of *J’* and *J* by using quantum Monte Carlo methods would also provide significant insight into the landscape of accessible states in such a related family of materials. This would enable such quantum phase transitions to be achieved in the better isolated spin ladders in (3,4‐lutH)_2_CuBr_4_, enabling these exotic magnetic phases to be probed in detail as required to develop a deeper understanding of these phenomena.

## Conclusions

This study concerns the synthesis of (3,4‐lutH)_2_CuBr_4_, which is found to have a crystal structure comprising well isolated bilayers with smaller coupling pathways between the two layers in a bilayer through Br⋅⋅⋅Br contacts than within them. Its magnetic susceptibility is consistent with a spin‐1/2 system and is well modelled by a Heisenberg model with dominant antiferromagnetic coupling between neighbouring dimers in the different layers of the bilayer and ferromagnetic coupling within a layer. DFT calculations are consistent with this, but suggest that magnetic coupling is only significant along one of the two directions within a layer; this would suggest that (3,4‐lutH)_2_CuBr_4_ resembles a magnetic spin ladder with ferromagnetic rail coupling. Experimental characterisation on the atomic scale is needed to distinguish whether this material is a magnetic bilayer or spin ladder but either would be a unique spin‐1/2 homometallic inorganic‐organic magnet.

## Experimental Section

CuBr_2_ was obtained from ACROS; all solvents and HBr were obtained from Fisher Scientific. All starting materials were used without further purification. To make single crystals of (3,4‐lutH)_2_CuBr_4_ a suspension of CuBr_2_ (0.5 g, 2.2 mmol) in EtOH (40 cm^3^) was made and HBr (9 M, 0.75 cm^3^, 6.75 mmol) added, followed by 3,4‐lutidine (0.5 cm^3^, 4.5 mmol). The muddy brown mixture was heated briefly to boiling and then allowed to cool to ambient temperature, giving a deep green solution. Crystals were grown by vapour diffusion of Et_2_O into the reaction mixture at ambient temperature over two days. The product was isolated by filtration and washed with cold EtOH (10 cm^3^) and cold Et_2_O (10 cm^3^) and dried under a flow of air, giving crude product (761 mg, ca. 1.3 mmol, ca. 59 % yield) as dark purple needles suitable for single‐crystal diffraction. The crude product was found to be impure by powder X‐ray diffraction and a phase pure sample was obtained by dissolving crude (3,4‐LutH)_2_CuBr_4_ (0.175 g) in EtOH (9 cm^3^) spiked with 3 drops of HBr (9 M), followed by vapour diffusion of Et_2_O; a crop of single crystals were obtained after 2 days (45 mg) were used in further studies.

The structure was solved by using Rigaku Oxford Diffraction Supernova Dual Source Diffraction with Cu_Kα_ (*λ*=1.54184 Å) radiation at 100 K and the sample mounted on MiTeGen microloops. Unit cell determination, data reduction and absorption corrections were carried out using CrysAlisPro 171.38.46.^[56]^ Using the Olex2 GUI,^[57]^ the structure was solved with the ShelXT structure solution program^[58]^ through Direct Methods and refined with the ShelXL refinement package^[59]^ using Least Squares minimisation. Non‐hydrogen atoms were refined anisotropically and hydrogen atoms were included using a riding model. All thermal ellipsoid plots were generated using CrystalMaker.^[60]^

Deposition Number 1959032 contains the supplementary crystallographic data for this paper. These data are provided free of charge by the joint Cambridge Crystallographic Data Centre and Fachinformationszentrum Karlsruhe Access Structures service.

Power X‐ray diffraction patterns were obtained using a Rigaku Miniflex using Cu_Ka_ (40 kV, 15 mA) equipped with a D/tex Ultra detector with the sample mounted on an aluminium sample plate.

Variable‐temperature direct current (DC) magnetic property measurements were carried out on (3,4‐lutH)_2_CuBr_4_ using a MPMS‐7 Quantum magnetometer while isothermal magnetisation measurements were carried out using a Quantum Design PPMS‐9. Alternating current (AC) measurements were performed using a Quantum Design MPMS XL‐7. In all cases the sample was held in a gelatin capsule mounted inside a pierced straw with a uniform diamagnetic background. Variable temperature data were collected in either settle mode or sweep mode at a rate no faster than 1 K/min. Thermogravimetric analysis (TGA) and differential thermal analysis (DTA) were carried out simultaneously using a NETZSCH 409 PG/PC TGA. The sample was held in an aluminium crucible and heated under flowing air at a rate of 10°/minute to 800 °C.

First principles calculations were carried out to gain further insight into the magnetic interactions in (3,4‐lutH)_2_CuBr_4_, particularly those within individual structural layers between which the magnetic property measurements could not distinguish. The DFT calculations employed the generalised gradient approximation (GGA) implemented with projector augmented‐wave (PAW)[^61^, ^62^] pseudopotentials as supplied in the Vienna Ab Initio Simulation Package (VASP)[^63^, ^64^] DFT calculation employed corrections for van der Waals dispersion interactions (optB86b‐vdW),[^65^, ^66^] and an on‐site Coulomb repulsion,^[67]^ U, was considered for the Cu 3d orbitals. A 2×2×1 Monkhorst–Pack k‐point mesh for the 82‐atom unit cell (appropriately scaled for supercells), and a 800 eV plane‐wave cut‐off energy were found sufficient to converge the total energy, forces and stresses within 0.5 meV/atom, 1 meV/Å and 0.5 kbar respectively.

## Conflict of interest

The authors declare no conflict of interest.

1

## Supporting information

As a service to our authors and readers, this journal provides supporting information supplied by the authors. Such materials are peer reviewed and may be re‐organized for online delivery, but are not copy‐edited or typeset. Technical support issues arising from supporting information (other than missing files) should be addressed to the authors.

Supporting Information

## Data Availability

The data that support the findings of this study are available from the corresponding author upon reasonable request.
