# Supplementary material for: Quantum Spin‐1/2 Dimers in a Low‐Dimensional Tetrabromocuprate Magnet
Source: Chemistry. 2022 Apr 27;28(32):e202200855. doi: 10.1002/chem.202200855 (PMC9323490; doi:10.1002/chem.202200855)
Supplement: Supplementary file 1 — Supporting Information [file CHEM-28-0-s001.pdf]

Chemistry–A European Journal

Supporting Information

Quantum Spin-$^1/_2$ Dimers in a Low-Dimensional Tetrabromocuprate Magnet

Gavin Sampson, Nicholas C. Bristowe, Sam T. Carr, Asad Saib, Gavin B. G. Stenning, Ewan R. Clark, and Paul J. Saines*

Single Crystal X-ray Diffraction

Table S1: Crystallographic data for $(3,4\text{-LutH})_2CuBr_4$ determined in this work by single crystal X-ray diffraction. DFT lattice parameters are shown in italics.

Compound	**$(3,4\text{-lutH})_2CuBr_4$**
Formula	$Cu\ C_{14}\ H_{20}\ Br_4\ N_2$
Formula Weight	599.50
T (K)	150(2)
λ (Å)	1.54184
Crystal System	Triclinic
Space Group	P1
a (Å)	7.7342(3)<br>*7.627*
b (Å)	8.1537(3)<br>*8.096*
c (Å)	15.9516(8)<br>*15.608*
α (°)	76.184(4)<br>*77.50*
β (°)	76.112(4)<br>*77.37*
γ (°)	88.726(3)<br>*90.19*
V (Å^3)	947.63(7)<br>*916.79*
Z	2
ρ_{cal} (g cm^{-3})	2.101
μ (cm^{-1})	11.458
Refl. meas./unique	5801/3462<br>[R_{int} = 0.0230]
Parameters refined	194
R_1, wR_2 (all)	0.0250, 0.0551
R_1, wR_2 (obs)	0.0225, 0.0538
Goodness of Fit	1.026

Table S2: Cu-Br bond and Br…Br contact distances and Br-Cu-Br bond angles for $CuBr_4$ tetrahedra in $(3,4\text{-lutH})_2CuBr_4$. DFT values are shown for comparison in italics.

Cu-Br<br>Br…Br	Distance (Å)	Br-Cu-Br	Bond Angle (°)
Cu1-Br1	2.3839(5)<br>*2.415*	Br1-Cu1-Br2	97.924(18)<br>*97.77*
Cu1-Br2	2.3853(5)<br>*2.406*	Br1-Cu1-Br3	132.04(2)<br>*130.84*
Cu1-Br3	2.4025(5)<br>*2.430*	Br1-Cu1-Br4	100.036(17)<br>*100.70*
Cu1-Br4	2.3704(5)<br>*2.392*	Br2-Cu1-Br3	98.839(17)<br>*99.17*
Br1…Br1	3.739(8)<br>*3.570*	Br2-Cu1-Br4	134.08(2)<br>*133.82*
Br1…Br3	4.408(7)<br>*4.303*	Br3-Cu-Br4	99.666(18)<br>*99.88*
Br2…Br4	4.429(5)<br>*4.258*		

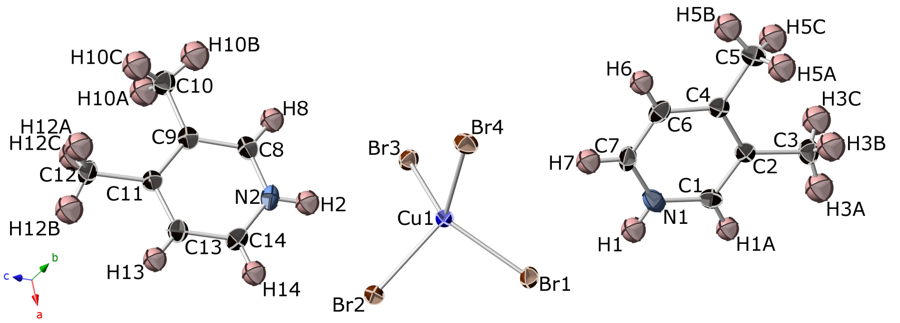

Figure S1: The asymmetric unit of the crystal structure of $(3,4\text{-lutH})_2CuBr_4$ with atoms displayed as thermal ellipsoids with 50 % probability. The Cu, bromide, carbon, nitrogen and hydrogen atoms shown as dark blue, maroon, black, light blue and pink spheres, respectively.

Powder X-ray Diffraction

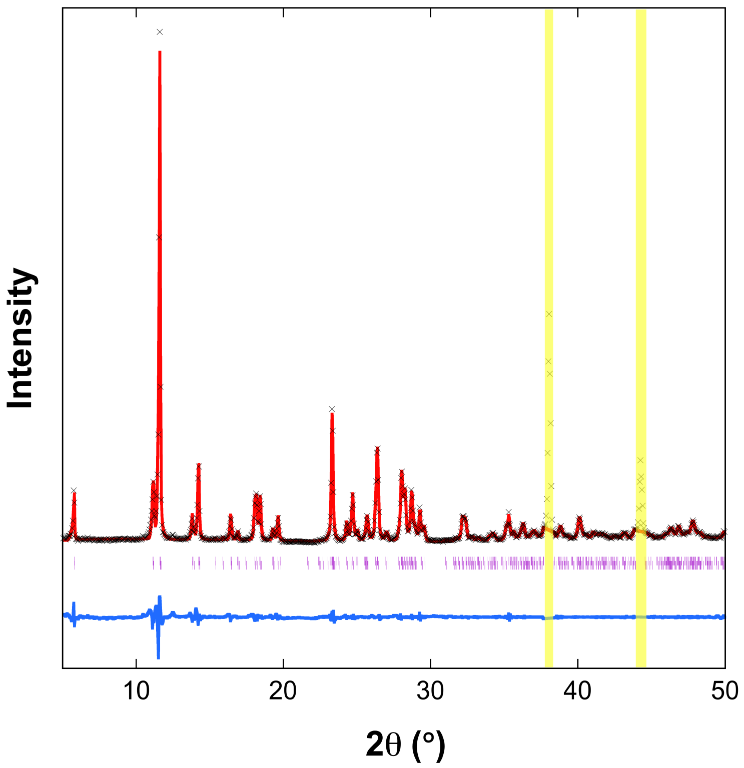

Figure S2: Le Bail fit to a diffraction pattern of a sample of $(3,4\text{-lutH})_2CuBr_4$. The dots, red line and blue line represent the experimentally observed and calculated intensities and the difference plot between them, respectively. Excluded regions are highlighted in yellow and include the two observed reflections for the Al sample holder. R_p, R_{wp} and χ^2 from the refinement are 4.23 %, 6.79 % and 9.49 and the unit cell parameters refined as a = 7.8382(7) Å**,** b = 8.2107(7) Å, c = 16.2736(9) Å, α = 75.355(7)°, β = 75.390(8)° and γ = 88.123(10)°, giving a unit cell volume of 980(3) Å^3, respectively.

Thermal analysis

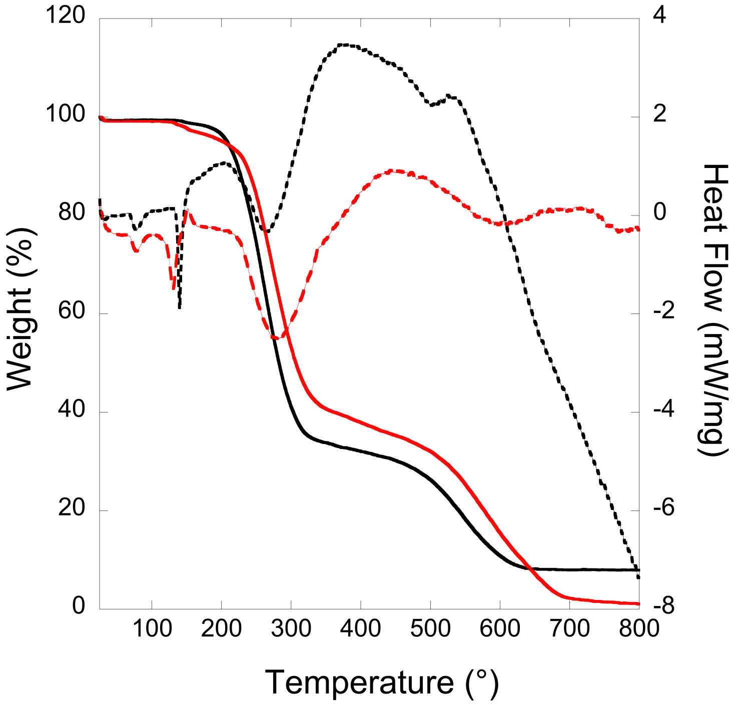

Figure S3: Thermogravimetric (solid lines) and differential thermal (dotted lines) analysis obtained from $(3,4\text{-lutH})_2CuBr_4$ in air (black) and nitrogen (red).

Magnetic Property Measurements

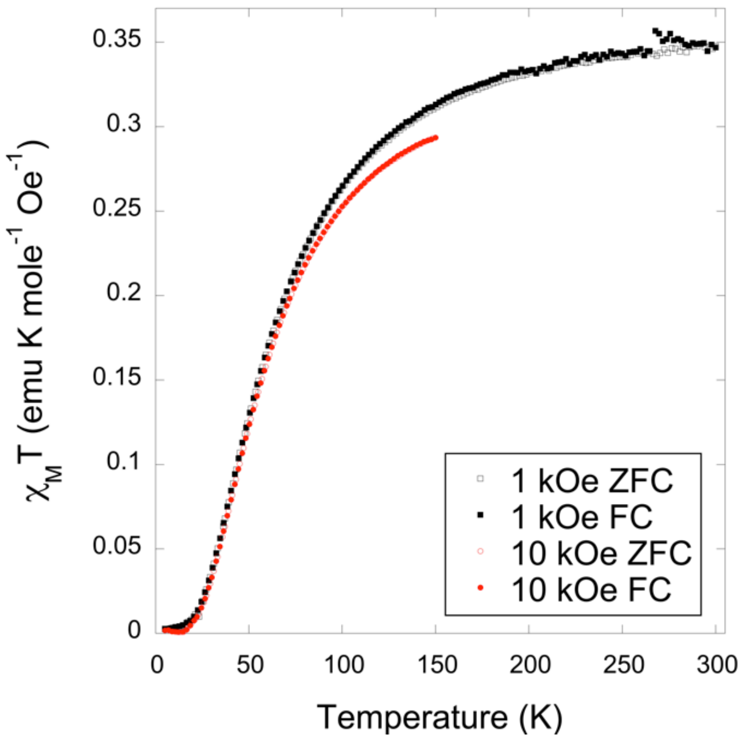

Figure S4: χT versus temperature for (3,4-lutH)$_2$CuBr$_4$ at 1 kOe and 10 kOe with ZFC and FC measurements in hollowed and filled symbols respectively.

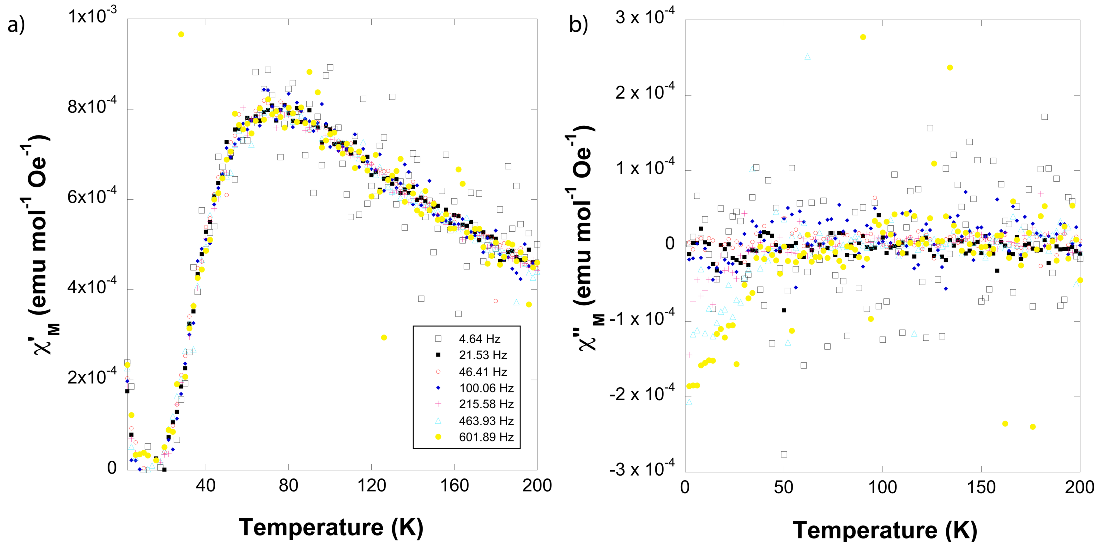

Figure S5: a) χ' and b) χ" measurements versus temperature with an applied AC field of 3 Oe at various frequencies in the absence of a DC field.

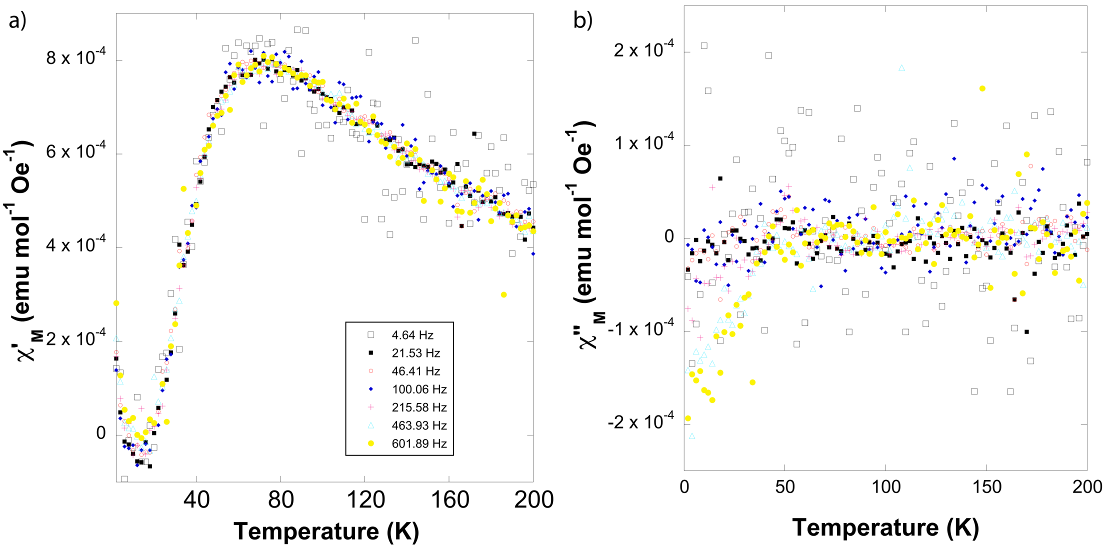

Figure S6: a) χ' and b) χ" measurements versus temperature with an applied AC field of 3 Oe at various frequencies under a constant DC field of 50 Oe.

Magnetic Model Derivation

The temperature dependence of χ for isolated magnetic dimers with Heisenberg spins is very well known (see Equation S1). A clear report of the derivation of the magnetic model used to fit the temperature dependence of χ in this work, namely that of magnetic dimers with significant inter-dimer coupling, is lacking. For completeness we present the derivation used in our work.

$$\chi_0 = \left.\frac{dM}{dB}\right|_{B=0} = \frac{1}{T}\frac{2}{3+e^{-J/T}} \qquad \text{Equation S1}$$

where χ_0, M, B, T and J indicate the magnetic susceptibility of an isolated dimer, the sample magnetisation, the magnetic flux density, the temperature and the strength of the coupling within a dimer (where a negative J indicates antiferromagnetic interactions).

The magnetisation of each dimer is:

$$M = \chi B$$

where χ is the magnetic susceptibility of each dimer.

The effective field seen on each dimer is:

$$\begin{aligned} B_{eff} &= B + \frac{J'}{2}M \\ &= B\left(1 + \frac{J'}{2}\chi\right) \end{aligned}$$

where B_{eff} is the effective magnetic field experienced by each dimer and $J'/2$ parameterises the strength of the interaction between dimers.

This gives:

$$\begin{aligned} M &= \chi_O B_{eff} \\ &= \chi_O B\left(1 + \frac{J'}{2}\chi\right). \end{aligned}$$

Therefore since $M = \chi B = \chi_O B\left(1 + \frac{J'}{2}\chi\right)$:

$$\chi - \frac{J'}{2}\chi_O\chi = \chi_O$$
$$\chi = \frac{\chi_O}{1 - J'\chi_O\chi/2}$$
$$= \frac{1}{\chi_0^{-1} - J'/2}.$$

Rearranging Equation 1 gives:

$$\chi_O^{-1} = \frac{T\left(3 + e^{-J/T}\right)}{2}.$$

Therefore since $\chi = \frac{1}{\chi_0^{-1} - J'}$:

$$\chi = \frac{1}{\left(T\left(3 + e^{-J/T}\right) - J'\right)/2}$$
$$= \frac{2}{T\left(3 + e^{-J/T}\right) - J'}$$
$$= \frac{1}{T}\frac{2}{3 + e^{-J/T} - J'/T}.$$

Parameterising g-factors, number of ions etc.:

$$\chi = \frac{4C/T}{3 + e^{-J/T} - J'/T}$$

where the factors are chosen so that at large T>>J, $\chi \sim \frac{C}{T}$ i.e. C is equivalent to the Curie constant at large temperature.

Adding an impurity contribution for an isolated spin obeying Curie-Weis law gives:

$$\chi = \frac{4C/T}{3 + e^{-J/T} - J'/T} + \frac{C_{imp}}{T - \theta_{imp}}$$

where C_{imp} and θ_{imp} are the Curie and Weiss constant of the impurity, respectively, as used to fit the temperature dependence of the observed magnetic susceptibility.

Within this mean field calculation $J'/2$ is the sum of all interactions between a dimer and its neighbours. This means in a nearest neighbour model that $J' = 2zJ_N$, where z is the number of nearest neighbours. This mean-field model neglects thermal and quantum fluctuations, which are particularly significant in low dimensional systems. This leads to the J' obtained from an experimental fit using this model to be an underestimation of the strength of the magnetic coupling between nearest neighbours between dimers. Despite the limitations in determining the precise values of J_N from fitting the derived model to the susceptibility data the overall shape of χ derived is robust to such fluctuations, as is the case in all mean field theory approximations including Curie-Weiss itself.

DFT magnetic ordering energetics

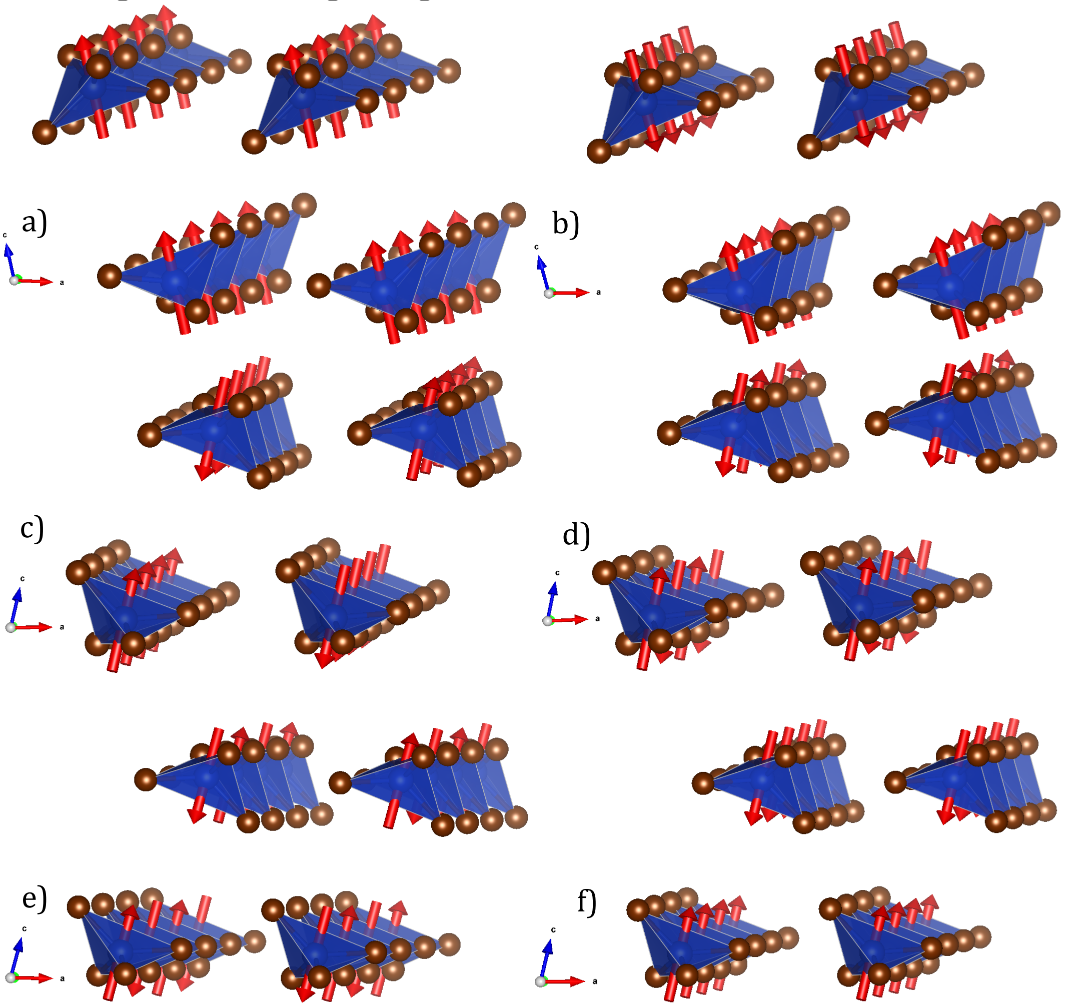

Figure S7: Depiction of the coupling within a bilayer for a) FM, b) AFM1, c) AFM2, d) AFM3, e) AFM4 and f) AFM5. For clarity only $CuBr_4$ tetrahedra are shown with the calculated coupling of the collinear magnetic spins shown arbitrarily along the *c*-axis since such models do not consider the spin direction. The coupling between bilayers along the *c*-axis is ferromagnetic for FM and AFM5 but is antiferromagnetic in all other cases considered.

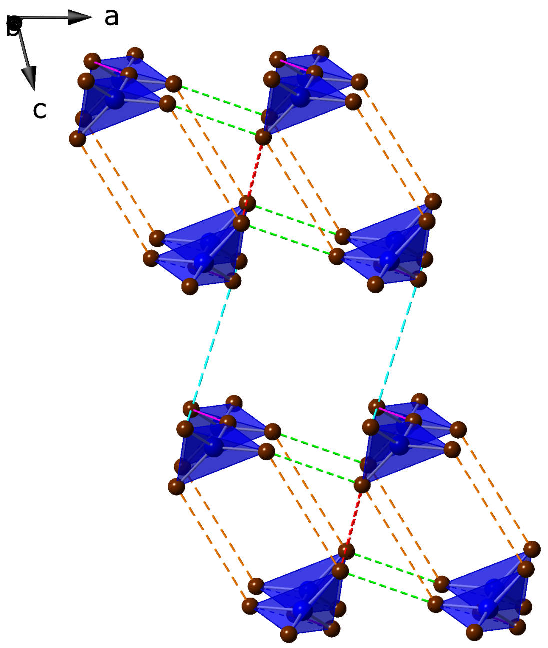

Figure S8: Depiction of the Br…Br contacts associated with each of the J values determined from DFT calculations. J, J_{N1}, J_{N2}, J_{N3} and J_{N4} are indicated as dotted red, green, pink, light blue and orange dotted lines, respectively. All other colours are the same as Figure 1 and, for clarity only the $CuBr_4$ polyhedra are shown.

Table S3: DFT total energies of six different magnetic orderings as a function of on-site Coulomb repulsion, U.

Magnetic Ordering	Spin exchange energy per unit cell	Energy relative to FM (meV per unit cell)				
		U=3eV	U=5eV	U=7eV	U=9eV	U=11eV
FM	$(-J -2J_{N1} -2J_{N2} -J_{N3} -J_{N4})/4$	0.0	0.0	0.0	0.0	0.0
AFM1	$(J -2J_{N1} -2J_{N2} +J_{N3} +J_{N4})/4$	-26.1	-20.4	-14.4	-8.2	-4.2
AFM2	$(J +2J_{N1} -2J_{N2} +J_{N3} -J_{N4})/4$	-0.4	-0.6	-0.8	-0.8	-0.9
AFM3	$(J -2J_{N1} +2J_{N2} +J_{N3} +J_{N4})/4$	-27.1	-21.5	-15.4	-9.2	-5.1
AFM4	$(J +2J_{N1} +2J_{N2} +J_{N3} -J_{N4})/4$	-1.9	-2.1	-2.2	-2.3	-2.3
AFM5	$(J -2J_{N1} -2J_{N2} -J_{N3} +J_{N4})/4$	-25.6	-20.2	-14.4	-8.3	-4.4
